# Common symptoms of Alzheimer’s dementia that are easily recognizable by families

**DOI:** 10.1590/1980-57642021dn15-020005

**Published:** 2021

**Authors:** Raden Siti Maryam, Junaiti Sahar, Sutanto Priyo Hastono, Kuntjoro Harimurti

**Affiliations:** 1 Faculty of Nursing, Universitas Indonesia - Jawa Barat, Indonesia.; 2 Faculty of Medicine, Universitas Indonesia - Jawa Barat, Indonesia.; 3 Faculty of Public Health, Universitas Indonesia - Jawa Barat, Indonesia.

**Keywords:** Alzheimer disease, dementia, family, memory disorders, doença de Alzheimer, demência, família, transtornos da memória

## Abstract

The increase in dementia incidence among the elderly is directly related to aging, which is associated with changes in bodily functions and other health-related risk factors. Alzheimer's dementia is the most prevalent form of dementia, and individuals in the late stages are predominantly dependent on other family members. Therefore, it is important for families, as the closest support group, to recognize common symptoms early.

**Objective:**

To provide a family-friendly guide to the ten common symptoms of Alzheimer's dementia.

**Methods:**

This is a descriptive survey-based research that included 354 families comprising elderly people (≥60 years) residing in Jakarta. The instrument aimed at identifying ten common Alzheimer’s dementia symptoms in Indonesia. Descriptive statistical analysis based on frequency tables was used.

**Results:**

The participant’s major characteristics were age ≥66 years (52.3%), female sex (70.3%) and primary school education (87.3%). The predominant symptoms experienced by 42.4% of the elderly included forgetting recent events and asking questions and narrating a particular detail repeatedly. The remaining 35.6% demonstrated signs of forgetting where an item was placed and frequently suspecting others of theft and concealment of personal items.

**Conclusion:**

The symptoms of frequently forgetting new events and the location of personal belongings are of particular concern for families, as they have a propensity to progress and interfere with daily activities. Therefore, the families of affected individuals are expected to identify this symptom early on and present the affected individual for screening or examination at a health care facility.

## RESUMO.

O aumento da incidência de demência entre os idosos está diretamente relacionado ao envelhecimento, que está associado a alterações nas funções corporais e outros fatores de risco relacionados à saúde. A demência de Alzheimer é a forma mais prevalente de demência e os indivíduos nos estágios avançados são predominantemente dependentes de outros membros da família. Portanto, é importante que as famílias, como grupo de apoio mais próximo, reconheçam os sintomas comuns precocemente.

**Objetivo:**

fornecer um guia familiar para os dez sintomas comuns da demência de Alzheimer. Métodos: Trata-se de uma pesquisa descritiva de base *survey* que incluiu 354 famílias compostas por idosos (≥60 anos) residentes em Jacarta. O instrumento teve como objetivo identificar dez sintomas comuns de demência de Alzheimer na Indonésia. Foi utilizada análise estatística descritiva baseada em tabelas de frequência.

**Resultados:**

As principais características do participante foram idade ≥ 66 anos (52,3%), sexo feminino (70,3%) e escolaridade primária (87,3%). Os sintomas predominantes vivenciados por 42,4% dos idosos incluem o esquecimento de acontecimentos recentes, fazer perguntas e narrar um determinado detalhe repetidamente. Os 35,6% restantes demonstraram sinais de esquecimento de onde um item foi colocado e frequentemente suspeitam de furto e ocultação de itens pessoais.

**Conclusão:**

Os sintomas de esquecimento frequente de novos eventos e da localização de objetos pessoais são de particular preocupação para as famílias, visto que apresentam tendência para progredir e interferir nas atividades diárias. Portanto, espera-se que as famílias dos indivíduos afetados identifiquem esse sintoma logo no início e apresentem o indivíduo afetado para triagem ou exame em uma unidade de saúde.

## INTRODUCTION

There has been a significant increase in the national elderly population of Indonesia within the last 8 years. The elderly comprised 7.56% of the total population of 238.5 million in 2010, and this percentage is projected to increase to 15.77% of a predicted total population of 305.6 million in 2035. Similarly, life expectancy, which was 70.1 years in 2010-2015, is expected to increase to 72.2 years in 2030-2035.^1^ A similar trend has been predicted for Jakarta, too, where the elderly population was 5.13% of the total population of 9.6 million in 2010 and is projected to increase to 16.39% of an expected total population of 11.5 million in 2035. Similarly, life expectancy in 2010-2015 was 71.6 years, and it is expected to increase to 73.9 years in 2030-2035.^1^ These data indicate a positive correlation between an increase in the elderly population and age.

The functional consequence theory proposes that there is a close association between increase in age and changes in bodily functions. These modifications in bodily functions are stimulated by the aging process, along with supplementary risk factors.^2^ In addition, this biological theory is explained by the decline in internal cellular function that leads to acceleration of tissue damage. Consequent functional changes are observed in the human body, including the nervous system, which is marked by a decline in cognitive processes such as thinking and remembering.^2^

Dementia is a syndrome in which there is deterioration in memory, thinking, behavior and the ability to perform everyday activities.^3^ ^,^ ^4^ ^,^ ^5^ ^,^ ^6^ ^,^ ^7^ ^,^ ^8^ ^,^ ^9^ There were approximately 46.8 million dementia patients worldwide in 2015, and this figure is expected to steadily increase to 74.7 million in 2030 and 131.5 million in 2050. In addition, statistics indicate an estimate of one new case per 3.2 seconds or 9.9 million per year, distributed worldwide as follows: 4.9 million in Asia, 2.5 million in Europe, 1.7 million in Americas and 0.8 million in Africa.^3^ Particularly, the prevalence in Indonesia reached 1.2 million people as of 2015, where a continuous increase up to 1.9 million is expected in 2030 and approximately 4 million is expected in 2050.^4^

Preliminary research in Jakarta has revealed a predominant elderly population (>65 years) that is at risk of dementia (p<0.05).^5^ A study in Yogjakarta also showed an increase in prevalence along with age: the incidence was 1 in 10 elderly individuals aged 60 years, 2 in 10 individuals aged 70 years, 4-5 in 10 individuals aged 80 years, and 7 in 10 elderly individuals aged 90.^6^ Additionally, various studies have shown that the dementia incidence doubles with every 6.3 years of age: it is 3.9 per 1000 people/year in the age group 60-64 years and 104.8 per 1000 people/year in the age group >90 years.^3^ However, awareness and understanding about this disease in Indonesia is poor, from the perspectives of both the elderly and caregivers. Only 4-16% of affected individuals can recognize the symptoms of dementia.^6^ ^,^ ^7^ Unfortunately, the social stigma associated with this condition presents obstacles in its diagnosis and care. Furthermore, it is considered an additional burden for the family, caregivers and the community from both a psychological and economic perspective.^6^

The elevated incidence of dementia worldwide and in Indonesia also has led to the development of global and national health policies about dementia. Accordingly, the WHO has launched a global action plan for public health response to dementia for 2017-2025. This plan includes: improving awareness and building a dementia-friendly environment; limiting the potential risks; providing diagnosis, treatment and support services for caregivers; building information systems; and conducting research as well as promoting innovations.^8^ In addition, efforts to reduce the risks of cognitive impairment have also been made^9^ through a national action plan that was executed as one of the nationwide control strategies against Alzheimer’s dementia.^10^ These prevention and management programs ought to be carried out early enough and commenced at the family level.

The data reported so far indicate the need for increased and focused efforts to prevent and control dementia in the elderly. This can be achieved by increasing awareness among family members about the ten common symptoms of Alzheimer’s dementia as the most common type of dementia. Therefore, the purpose of this study was to describe the ten common symptoms of Alzheimer’s dementia in the elderly, which will help family members recognize its symptoms early and take the necessary precautions and actions.

## METHOD

This is a survey-based descriptive study that included 354 families. The families met the inclusion criteria, which were having elderly members aged ≥60 years and living in the same house in the Jakarta area in Indonesia.

The sampling process was divided into the following stages based on geographical location: First, from each of the five regions in Jakarta, participants were selected from a subdistrict with the largest amount of older adults. Second, from each subdistrict, a village with the largest number of older adults was selected. Third, a Rukun Warga (Citizen Association) was selected from each village. Fourth, families with elderly were selected based on proportion. Therefore, the total sample included 354 families.

A questionnaire was used to obtain information about demographic characteristics, such as age, gender, education status, occupation and the number of elderly people. The instrument used to assess knowledge about the ten common symptoms was obtained from Alzheimer’s Indonesia and was verified based on a Cronbach’s α value of 0.8.

Data processing and analysis were performed using SPSS 20, and the results were presented in tabular form. The data distribution for each variable was described according to the respective characteristics of the family and the elderly. In addition, the general symptoms experienced were evaluated in terms of frequency and percentage.

This study was reviewed and approved by the Ethics Committee, Faculty of Nursing, University of Indonesia (Approval No. 46/UN2.F12.D/HKP.02.04/2018), and adherence to the three basic ethical principles, namely respect for each individual, beneficence and justice, was ensured. Therefore, the study was initiated with an explanation of the objectives, and the respondent’s consent was obtained by voluntary signing of the form. All data were kept confidential and used for research purposes only.

## RESULTS

Table 1 shows data on prevalence, indicating that 52.8% of families with elderly possess low education, followed by careers, where most, about 15.5%, are laborers, and 72.9% are low income earners.

**Table 1.** Characteristics of families caring for the elderly.

Family characteristics	Frequency (percentage)
Age	
Child (≤14 y) Adolescent (15-21 y) Adult (22-59 y) Fellow elderly (≥60 y)	1 (0.3) 19 (5.4) 294 (83.1) 40 (11.3)
Gender	
Male Female	79 (22.3) 275 (77.7)
Education level	
Primary school Secondary school Tertiary school	187 (52.8) 137 (38.7) 30 (8.5)
Occupation	
Farmer Laborer Employee at a private firm Civil servant Entrepreneur Other	1 (0.3) 55 (15.5) 53 (15.0) 2 (0.6) 52 (14.7) 1 (0.3)
Income	
Less than Provincial Minimum Wage (UMP) More than Provincial Minimum Wage (UMP)	258 (72.9) 62 (17.5)

UMP: Upah Minimum Provinsi DKI Jakarta for 2017 is around 3.355.750 IDR per month.

Table 2 show data the elderly, aged over 66 years, tend to have a higher prevalence (reaching 52.3%), where most are women (70.3%), and their education level was low, reaching 87.3%.

**Table 2.** Characteristics of the elderly in the included families.

Characteristics	Frequency (percentage)
Age	
<66 y ≥66 y	169 (47.7) 185 (52.3)
Gender	
Male Female	105 (29.7) 249 (70.3)
Education level	
Primary school Secondary school Tertiary school	309 (87.3) 41 (11.6) 4 (1.1)

Table 3 show data the most common symptoms of dementia-Alzheimer in the elderly include memory loss, reaching 42.4%, followed by being forgetful, misplacing stuff (35.6%) and the persistence of mood swings, emotional changes (31.9%).

**Table 3.** Frequency distribution of 10 common symptoms of Alzheimer’s dementia in the elderly by family.

No	10 common symptoms of Alzheimer’s dementia	Frequency (percentage)	
		No symptoms appear	Symptoms appear (highest to lowest incidence)
1	The elderly often forget events that have just happened, and ask questions and say the same things repeatedly	204 (57.6)	150 (42.4)
2	The elderly forget where they put something, and even suspect that someone has stolen or hidden their items	228 (64.4)	126 (35.6)
3	The elderly’s emotions change drastically: they become confused, suspicious, afraid, overly dependent on family members and easily frustrated and discouraged, both at home and at work	241 (68.1)	113 (31.9)
4	The elderly often find it difficult to plan or complete daily tasks, to drive and to manage finances	247 (69.8)	107 (30.2)
5	The elderly find it difficult to perform daily activities and work, forget how to cook and operate the telephone/cell phone, cannot do simple calculations and work longer hours than usual	253 (71.5)	101 (28.5)
6	The elderly are confused about time (important days/dates) and where they are, and do not know the way back home	266 (75.1)	88 (24.9)
7	The elderly have difficulty in speaking and finding the right words, and often stop in the middle of a conversation and are confused about how to continue	281 (79.4)	73 (20.6)
8	The elderly do not have the enthusiasm or initiative to carry out activities or hobbies that they usually enjoy with their friends	282 (79.7)	72 (20.3)
9	The elderly find it difficult to coordinate their clothes when dressing (for example, colors that are not compatible are worn together), are unable to count money correctly when purchasing something, and cannot take good care of themselves	308 (87)	46 (13)
10	The elderly find it difficult to judge distances, run into mirrors while walking, do not recognize their own faces, spill water because they cannot aim correctly when filling a glass, and find it difficult to distinguish colors	309 (87.3)	45 (12.7)

## DISCUSSION

According to this survey, families with elderly are mostly characterized by low education (52.8%) This finding is congruent with previous research (reporting 47.9% families with low education) which has shown that the level of education and income can influence readiness towards caring for elderly individuals.^11^ Accordingly, the knowledge aspect is one of the factors known to influence the formation of thought patterns, attitudes and behavioral changes.^12^

The present results indicate that the predominant family occupation was laborer, and the income was below the Provincial Minimum Wage in the majority (72.9%) of the families. This finding is in line with the outcome of a study that demonstrated the impact of economic status on health services in terms of treatment cost, health insurance participation and coverage of health care facilities.^11^ A previous study also showed a significant correlation of depression with cognitive impairment and economic level (p<0.05).^16^ Moreover, the finding is supported by a study that indicated a correlation between stressed families and the realized need for more help.^20^

As per cultural practices in Indonesia, the extended family model is still strong and potentially instigates feelings of greater comfort, care and a sense of belonging between the elderly and other family members. This situation serves as the basis for health workers to provide families with counselling.^13^ ^,^ ^17^ However, many aging individuals and the designated caregivers consider cognitive impairment as a normal phenomenon in old age.^2^ This finding is congruent with research stipulating the importance of knowledge related to dementia in making decisions, especially with regard to determining the appropriate treatment plan.^19^ In addition, it is essential to promptly recognize the symptoms and make an effort to control the risk factors.

With respect to gender, the incidence was higher in women, who comprised 70.3% of the affected population. The finding might be explained by secretion of the hormone oestrogen in women, as it may potentially improve their life expectancy in comparison with men. Additionally, 52.3% of the participants were over 66 years. Accordingly, other data indicate a positive correlation between increasing age and dementia prevalence among the elderly.^6^

The present results show a high incidence of low-level education (87.3%). Accordingly, previous research in Yogyakarta reports the incidence of low-level education to be 56%. However, this parameter has been found to have a significant negative correlation with the incidence of dementia or memory disorders in the PSTW (Panti Sosial Tresna Werdha).^14^

The incidence of memory disorders is one of the symptoms frequently observed. In addition, the survey showed that the tendency to forget recent events and ask questions and narrate similar details repeatedly was observed in 150 elderly individuals (42.4%). Furthermore, the survey showed that 126 participants (35.6%) were unable to remember where they had placed items. This finding confirms previous observations of the tendency to experience dementia after a recent event; forget where keys, wallets or papers are placed; experience difficulty remembering important phone numbers, dates and appointments.^14^

In the present survey, 28.5% of the elderly experienced difficulties while performing specific daily activities (such as cooking), operating the telephone and simple calculations, and displayed a tendency to work longer hours. This percentage is more than two times higher than the previously reported percentage of 11.8% with regard to difficulty in performing tasks that involve the use of instruments such as the telephone.^14^

In terms of self-care, the survey results showed difficulty in planning or completing daily tasks, confusion on how to drive and challenges in managing finances, with the cumulative percentage reaching 30.2%. The prevalence of these difficulties is higher than that reported in previous research, for example, difficulty with the use of a vehicle (3.9%), difficulty in managing finances (3.9%) and struggling with performing light housework (5.9%).^14^

With regard to the sixth most common symptom, that is, confusion about the day or date, uncertainty about current location and poor knowledge of the way back home, the incidence was 24.9%. This is lower than the incidence reported in previous studies, where the incidence of cognitive impairment with respect to time and place, as well as memory, as assessed using MMSE, was collectively 46.1%.^14^

Insufficient social interaction has been proposed to cause a decline in cognitive function.^15^ In the present survey, 20.6% of the elderly expressed difficulty in speaking and utilizing appropriate words, and therefore, often stopped in the middle of a conversation. Research also indicates the positive impact of changes in social interactions on the prevention of dementia.^15^

In this survey, 13% of the participants reported problems with coordinating clothes, paying the correct amount of money for purchasing an item and personal care. This is higher than the data reported in a prior study: 4.9% reported difficulty in counting money, 6.9% struggled with washing clothes and 1% had difficulty in regulating their medication use.^14^

A decrease in enthusiasm and initiative to perform activities or hobbies that were previously enjoyed with friends was also found in this survey. These symptoms can lead to depression in the elderly. A previous study also showed a significant correlation of depression with cognitive impairment and economic level (p<0.05).^16^

Poor knowledge about dementia symptoms among caregivers has been reported, especially with regard to recognizing difficulties faced by affected individuals in placing items in their appropriate place.^6^ The present findings also indicate difficulty in determining distance (as the participants reported that the elderly ran into mirrors while walking) and facial recognition. Additionally, unable to aim correctly when filling a glass with water and challenges with distinguishing colors were reported by 12.7% of the participants. This outcome is in line with previous findings that showed the influence of burnout syndrome on caregivers’ quality of life.^21^

Drastic emotional disturbances are observed in the elderly who are affected. This is prominently expressed as confusion, suspiciousness, fear, over-dependence and feeling easily disappointed and discouraged, either at home or at work, which were reported by 31.9% of the participants in this survey. However, depression, anxiety and apathy are common symptoms in sufferers, and the disease is known to influence mood as well as interest in daily activities and in people as well. This outcome is attributed to modifications in the brain that instigate an emotional reaction to a recent event.^18^

The present study’s description of ten common symptoms of Alzheimer’s dementia recognized by families in their elderly demonstrates the importance of health education in relation to dementia and its prevention, with the aim of increasing awareness and family care, given the impact of dementia from the physical, psychological, social and economic perspectives.

One of the limitations of this study was that its scope was restricted to only a general description of the ten Alzheimer’s dementia symptoms in the elderly. In addition, the family response and behavior after positive identification of symptoms were not studied.

To conclude, on the basis of the findings of this survey, educational and economic factors are expected to influence thought patterns, attitudes and behavior with regard to understanding common dementia symptoms and providing care for the elderly. Dementia is not a normal component of the aging process. However, memory loss is a common symptom that is predominant among the elderly and is expected to be of particular concern to families. The family being the smallest unit of society, is anticipated to assist in early discovery of dementia and to serve as an important prevention support system. Additionally, families play an important role in introducing elderly family members to health services for further examinations. These primary care services are expected to provide home visits and perform relevant screening, along with providing health education.
